# Comparative effectiveness of subcutaneous GLP-1 receptor agonists versus SGLT2 inhibitors on metabolic and hepatic outcomes in T2DM with MASLD: a propensity score-matched study

**DOI:** 10.1007/s40618-026-02976-3

**Published:** 2026-07-13

**Authors:** Barbara Toffoli, Matteo Sirignano, Valeria Bonato, Giulia Fusaro, Erica Russo, Valerio Velardi, Lory Saveria Crocè, Bruno Fabris, Riccardo Candido, Stella Bernardi

**Affiliations:** 1 Department of Medical Surgical and Health Sciences, University of Trieste, Ospedale Di Cattinara 447, 34149 Trieste, Italy; 2 Centro Clinico Studi Fegato ASUGI, Maggiore Teaching Hospital, Piazza Dell’Ospitale, 34100 Trieste, Italy; 3 SC Patologie Diabetiche, ASUGI (Azienda Sanitaria Universitaria Giuliano Isontina), 34128 Trieste, Italy; 4 SS Endocrinologia – Medicina Clinica, ASUGI, Ospedale Di Cattinara 447, 34149 Trieste, Italy

**Keywords:** GLP-1RA, SGLT2i, LIVER, MASLD, ALT, rs761387, SNP, T2DM

## Abstract

**Aims:**

Metabolic dysfunction-associated steatotic liver disease (MASLD) frequently coexists with type 2 diabetes mellitus (T2DM), complicating its management. In this population, we aimed to compare the effectiveness of subcutaneous GLP-1 receptor agonists (scGLP-1RA) to SGLT2 inhibitors (SGLT2i) on metabolic and hepatic parameters, while investigating the influence of *GLP1R* and *PNPLA3* genetic variants on treatment response.

**Methods:**

This retrospective study included patients with T2DM starting scGLP-1RA or SGLT2i, balanced through Propensity Score Matching (PSM) to account for baseline clinical differences. Clinical and biochemical data were compared at baseline and after one year of treatment. Genotyping was performed for *PNPLA3* (rs738409) and *GLP1R* (rs761387, rs6923761) polymorphisms. Multivariable linear regression was used to identify independent predictors of HbA1c, BMI, and transaminase reduction.

**Results:**

Both drug classes significantly improved glycemic control and BMI. However, scGLP-1RA demonstrated superior efficacy in HbA1c and BMI reduction compared to SGLT2i (β = -0.349, p = 0.001; β = -0.711, p = 0.045). In MASLD subgroups (baseline ALT > 35 U/L or hepatic steatosis), only scGLP-1RA led to a significant reduction in ALT levels (p = 0.02). Multivariable analysis identified the *GLP1R* rs761387 G allele as an independent predictor of a blunted glycemic response (β = -0.277, p = 0.018).

**Conclusions:**

In a real-world setting, scGLP-1RA showed higher potency than SGLT2i on metabolic and hepatic parameters. These findings support the preferential use of scGLP-1RA in patients with T2DM and MASLD and are consistent with current international recommendations. The association between *GLP1R* rs761387 variant and HbA1c reduction sheds light on the impact of this variant on treatment response. Further studies are needed to validate this genetic predictor in broader populations.

## Introduction

Metabolic dysfunction-associated steatotic liver disease (MASLD)[1] has emerged as the most prevalent chronic liver disorder worldwide, affecting more than 30% of the general population [2]. Its relationship with type 2 diabetes mellitus (T2DM) is fundamentally bidirectional and synergistic. On the one hand, T2DM acts as a potent driver for MASLD progression toward inflammation, fibrosis, and cirrhosis. According to a 2019 meta-analysis by Younossi, the global prevalence of MASLD among patients with T2DM is 55.5%, with metabolic dysfunction-associated steatohepatitis (MASH) affecting 37.3% of them [3, 4]. On the other hand, the presence of liver steatosis is associated with a 2.2-fold increased risk of incident diabetes, which parallels the underlying severity of MASLD [5]. In line with this, a recent 2023 consensus redefined liver steatosis to emphasize the critical role of metabolic dysregulation and placed T2DM at the core of its diagnostic criteria [1].

Despite the high prevalence of MASLD in patients with T2DM [3], therapeutic options specifically labeled for liver protection remain limited, and currently no glucose-lowering medication is formally approved for the treatment of MASLD [6]. However, glucagon-like peptide-1 receptor agonists (GLP-1RA) have shown promising results. In a phase 2 randomized controlled trial (RCT) involving 320 participants with MASH (65% of whom had T2DM), treatment with once-daily subcutaneous semaglutide (0.1–0.4 mg) for 72 weeks resulted in MASH resolution without worsening of fibrosis in 59% of individuals, compared to 17% in the placebo group [7]. More recently, in the phase 3 ESSENCE trial, which included 800 participants with MASH receiving semaglutide 2.4 mg once weekly for 72 weeks, fibrosis improvement was achieved in 36.8% of participants vs. 22.4% on placebo, while resolution of steatohepatitis occurred in 62.9% of participants vs. 34.3% on placebo [8]. Consistent with these findings, a recent consensus report from the American Diabetes Association [6] suggests that in patients with T2DM and steatotic liver disease preference should be given to GLP-1RA and/or GIP/GLP-1RA due to their efficacy in treating both hyperglycemia and steatohepatitis.

Nevertheless, sodium-glucose cotrasporter-2 (sGLT2) inhibitors should also be considered a valuable therapeutic option. For instance, in dedicated RCTs using non-invasive liver parameters as primary outcomes, SGLT2 inhibitors such as dapagliflozin, empagliflozin, or canagliflozin have been shown to significantly decrease hepatic steatosis [9, 10]. Regarding liver histology—specifically MASH resolution and fibrosis improvement—a recent large multicenter RCT has just demonstrated that dapagliflozin can achieve MASH resolution in 23% of patients vs 8% on placebo and fibrosis improvement in 45% of participants vs 20% on placebo [11].

When evaluating treatment response, it should be taken into account that there is a significant inter-individual variability, which may be partly explained by the patient’s genetic background. With respect to liver steatosis, the *PNPLA3* rs738409 polymorphism is associated with an increased risk of MASLD, more severe liver histology, and future development of hepatocellular carcinoma and cirrhotic complications [1, 12, 13]. In addition, Urias et al. demonstrated that among individuals receiving the GLP-1RA semaglutide, transaminase levels decreased more in patients with *PNPLA3* risk alleles [14]. Furthermore, emerging evidence suggests that variants of the GLP-1 receptor (*GLP1R*) gene, such as rs6923761 and rs761387, might modulate the individual sensitivity to GLP-1RA therapy, potentially affecting not only glycemic control but also hepatic outcomes [15].

To date, head-to-head real-world studies comparing the hepato-protective effects of SGLT2i and GLP-1RA, while accounting for these specific genetic predispositions, are lacking. Therefore, the aim of this retrospective study was to evaluate the impact of GLP-1RA versus SGLT2i treatment on the metabolic and hepatic profile of patients with T2DM and MASLD, and explore the potential role of *PNPLA3* and *GLP1R* genetic variants in modulating drug response.

## Materials and methods

### Study design

This is a retrospective cohort study aiming to investigate the impact of (i) subcutaneous GLP-1RA (scGLP-1RA) vs SGLT2i and (ii) *PNPLA3* rs738409C > G, *GLP1R* rs6923761G > A, and *GLP1R* rs761387A > G gene variants on the metabolic and hepatic profile in patients with T2DM and MASLD. Primary endpoints included glycated hemoglobin (HbA1c) reduction, body mass index (BMI) reduction, and the decrease in aspartate aminotransferase (AST) and alanine aminotransferase (ALT). This study was conducted in accordance with the Declaration of Helsinki, and the protocol was approved by the Institutional Review Board of the University of Trieste, Trieste, Italy (University of Trieste Comitato Etico di Ateneo #136 date 30/11/2023). Its design follows the recommendations of the Strengthening the Reporting of Observational Studies in Epidemiology (STROBE) Statement [16].

Eligible participants were adult patients (age > 18 years) with T2DM who were followed at the ASUGI Diabetes Center (S.C. Patologie Diabetiche, ASUGI, Trieste, Italy) and who had been treated with scGLP-1RA or SGLT2i for at least 12 months. Subcutaneous GLP-1RA included dulaglutide, liraglutide, and semaglutide, while SGLT2i included canagliflozin, dapagliflozin, and empagliflozin. Outcomes were analyzed by pooled pharmacological class. Exclusion criteria were: (i) patients under the age of 18; (ii) absence of follow-up data; (iii) non-adherence to the prescribed medication; (iv) diagnosis of type 1 diabetes mellitus or gestational diabetes; and (v) lack of informed consent to participate in the study. Based on these criteria, between December 2023 and April 2025 we consecutively identified 230 patients, who were fully informed on the purpose of the research and gave their written informed consent for inclusion in this study.

We collected data from electronic medical records regarding demographic characteristics, disease duration, medication (withdrawn and/or associated, if any), length of follow-up (from the introduction of GLP-1RA or SGLT2i to the last visit), diabetes-related complications (diabetic nephropathy, retinopathy, neuropathy and cardiovascular comorbidities). For paired analyses, we collected baseline (before treatment) BMI, HbA1c, complete blood count, AST, ALT from electronic medical records and measured them after the treatment period. The noninvasive assessment of the fibrosis-4 (FIB-4) index was calculated as follows: [age (years) × AST]/[platelet × ALT^1/2^]. All the participants underwent a liver ultrasound (US) once during the course of therapy (after 12 months). Liver stiffness measurement was performed with point shear wave elastography (Affinity 70, ElastPQ, Philips Healthcare, Amsterdam, The Netherlands).

### *GLP1R* and *PNPLA3* polymorphism

Among the subjects that were identified, 230/230 patients consented to genetic analyses. In these patients, a blood sample was collected for the analysis of *GLP1R* and *PNPLA3* SNPs. Genotyping was performed on peripheral blood mononuclear cells (PBMCs), as previously described [17, 18]. The selected polymorphisms (*GLP1R* rs6923761, *GLP1R* rs761387*, PNPLA3* rs738409) were genotyped using a real-time TaqMan allelic discrimination assay (TaqMan SNP Assay; ThermoFisher Scientific Waltham, MA USA). The Assay IDs were: C__25615272_20 (rs6923761); C______9616_10 (rs761387); and C______7241_10 (rs738409), respectively. The distribution of allelic frequencies was analyzed by a χ^2^ test to assess for potential deviation from the Hardy–Weinberg equilibrium.

### Statistical analysis

All statistical analyses were performed using the software “R” for statistical computing (version 4.4.0; R Development Core team, The R Foundation for Statistical Computing). Statistical significance was defined as p-values < 0.05. Continuous variables were reported as median with interquartile range (IQR) and the Shapiro–Wilk test was used to check for distribution normality. Categorical variables were expressed as absolute frequencies and percentages. Continuous variables were compared using the Mann–Whitney test or the Kruskal–Wallis test depending on the number of groups. Paired analysis (baseline – last follow up) was performed with the Wilcoxon signed-rank test. Categorical variables were compared using the chi-square test or Fischer’s exact test whenever appropriate. To compare patients treated with scGLP-1RA to patients treated with SGLT2i and reduce potential bias, a 1:1 ratio Propensity Score Matching (PSM) analysis was performed using the nearest neighbor method with a caliper of 0.20 (R package MatchIt). Paired analyses compared clinical (BMI) and biochemical data (HbA1c, AST, ALT, FIB-4) at baseline and after treatment. The patients were matched 1:1 by age, gender, baseline BMI and baseline HbA1c. To analyze any association between drug choice or gene variants and the response to therapy multivariate linear regression models were performed. In these models, the dependent variables were the reduction in HbA1c, BMI, AST and ALT. Models were adjusted for age, sex, baseline HbA1c, baseline BMI, type of medication (scGLP-1RA or SGLT2i), gene variants.

## Results

### Population characteristics

Overall, 230 patients with T2DM were identified. The median follow-up period was 17 months (IQR 14; 19). The study population had a median age of 69 years (IQR 62; 76), was predominantly male (63%, 144/230), and had a median BMI of 29.4 kg/m^2^ (IQR 25.8; 34.3). At baseline, the median HbA1c was 6.8% (IQR 6.3; 7.4), with a median diabetes duration of 3.5 years (IQR 1; 6). Hepatic steatosis was present in approximately 67% of the cohort (143/230), with a median Hamaguchi score of 2 (IQR 2; 4) and a median FIB-4 score of 1.45 (IQR 1.04; 1.97). Regarding liver stiffness measurement (n = 143), the majority of patients exhibited values < 5.0 kPa (68.2%) or between 5.1–9.0 kPa (28.7%), while only 3.2% scored higher. Baseline median ALT and AST levels were 23 U/L (IQR 16; 32) and 21 U/L (IQR 18; 27), respectively.

When comparing the treatment groups, 108 patients received scGLP-1RA and 122 received SGLT2i. Table 1 details the exact types of agents used, their distribution within the study population, and their respective maintenance dosages for both groups.

**Table 1:** Type of molecule and maintenance dosage before and after PSM

Variables	Before the PSM	After the PSM
	**scGLP-1RA (N = 108)**	**scGLP-1RA (N = 74)**
Dulaglutide, *n (%)*	42/108 (38.9%)	34/74 (45.9%)
0.75 mg	16/42 (38.1%)	15/34 (44.1%)
1.5 mg	26/42 (61.9%)	19/34 (55.9%)
Liraglutide, *n (%)*	3/108 (2.8%)	3/74 (4.1%)
1.2 mg	2/3 (66.7%)	2/3 (66.7%)
1.8 mg	1/3 (33.3%)	1/3 (33.3%)
Semaglutide, *n (%)*	63/108 (58.3%)	37/74 (50%)
0.25 mg	5/63 (7.9%)	3/37 (8.1%)
0.5 mg	15/63 (23.8%)	9/37 (24.3%)
1 mg	43/63 (68.3%)	25/37 (67.6%)
	**SGLT2i (N = 122)**	**SGLT2i (N = 74)**
Canagliflozin, *n (%)*	21/122 (17.2%)	12/74 (16.2%)
100 mg	15/21 (71.4%)	7/12 (58.3%)
300 mg	6/21 (28.6%)	5/12 (41.7%)
Dapagliflozin, *n (%)*	63/122 (51.7%)	42/74 (56.8%)
10 mg	63/63 (100%)	42/42 (100%)
Empagliflozin, *n (%)*	38/122 (31.1%)	20/74 (27.0%)
10 mg	25/38 (65.8%)	14/20 (70.0%)
25 mg	13/38 (34.2%)	6/20 (30.0%)

Patients receiving scGLP-1RA and patients receiving SGLT2i significantly differed at baseline regarding age, sex, BMI, HbA1c, and the prevalence of diabetes-related complications (Table 2).

**Table 2:** Population characteristics before and after PSM

Variables	Before the PSM			After the PSM		
	scGLP-1RA (N = 108)	SGLT2i (N = 122)	p-value	scGLP-1RA (N = 74)	SGLT2i (N = 74)	p-value
Age (years)	66 (59; 75)	72 (65; 77)	** < 0.0001**	68 (63; 76)	71 (64; 75)	0.517
Sex (male), *n (%)*	57 (52.8%)	87 (71.3%)	**0.003**	44 (59.5%)	43 (58.1%)	0.867
Disease duration (years)	4 (2; 10)	3 (1; 10)	0.315	6 (2;12)	2 (1;4)	** < 0.001**
Diabetes complications, *n (%)*	53 (49.1%)	84 (68.9%)	**0.002**	42 (56.8%)	46 (62.2%)	0.503
Metformin, *n (%)*	74 (68.5%)	70 (57.4%)	0.081	45 (60.8%)	44 (59.5%)	0.867
Diabetic Nephropathy, *n (%)*	9 (8.3%)	31 (25.4%)	** < 0.001**	7 (9.5%)	16 (21.6%)	**0.041**
Stroke, *n (%)*	0 (0%)	7 (5.7%)	**0.011**	0 (0%)	2 (2.7%)	0.155
Ischemic heart disease, *n (%)*	16 (14.8%)	30 (24.6%)	0.064	15 (20.3%)	15 (20.3%)	1
Diabetic Neuropathy, *n (%)*	13 (12%)	22 (18%)	0.206	10 (13.5%)	13 (17.6)	0.496
Diabetic Retinopathy, *n (%)*	4 (3.7%)	8 (6.6%)	0.331	4 (5.4%)	6 (8.1%)	0.512
Carotid plaques, *n (%)*	40 (37%)	56 (45.9%)	0.174	33 (44.6%)	30 (40.5%)	0.618
Baseline BMI (kg/m^2^)	33.1 (27.9; 37.9)	28.3 (25; 30.9)	** < 0.0001**	30.5 (24.9; 34.3)	28.9 (25.8; 33.8)	0.793
Baseline HbA1c (%),	6.9 (6.4; 7.6)	6.6 (6.1; 7.3)	**0.011**	6.8 (6.4; 7.4)	6.6 (6.2; 7.4)	0.306
Baseline AST (IU/L)	21 (18; 27)	21 (17; 27)	0.638	21 (18; 26)	21 (16; 27)	0.743
Baseline ALT (IU/L)	24 (17; 39)	22 (16; 29)	0.103	23 (15; 39)	22 (16; 30)	0.514
Baseline FIB-4 score	1.17 (0.91; 1.70)	1.66 (1.18; 2.15)	** < 0.0001**	1.38 (1.03; 2.15)	1.50 (1.13; 2.00)	0.079
Steatosis, *n* (%)	81 (77%)	62 (57.4%)	**0.002**	50 (69.4%)	45 (67.2%)	0.773
Hamaguchi score	3 (2; 4)	2 (2; 3)	**0.023**	3 (2; 4)	2 (1; 3)	0.131
Stiffness, n (%)			0.341			0.460
< 5 kPa	56 (69.1%)	42 (67.8%)		36 (72.0%)	33 (73.4%)	
5.1—9 kPa	24 (29.7%)	17 (27.4%)		14 (28%)	10 (22.2%)	
9.1 – 13 kPa	0 (0%)	2 (3.2%)		0 (0%)	1 (2.2%)	
13.1 – 17 kPa	1 (1.2%)	0 (0%)		0 (0%)	0 (0%)	
> 17 kPa	0 (0%)	1 (1.6%)		0 (0%)	1 (2.2%)	

Data are reported as median and IQR. The p-values refer to the comparison between scGLP-1RA and SGLT2i groups before and after PSM.

BMI, body mass index; HbA1c, glycated haemoglobin; AST, aspartate aminotransferase; ALT, alanine aminotransferase; FIB-4, Fibrosis-4 index

### Comparison of GLP1-RA vs SGLT2i on metabolic and liver parameters

**Fig. 1:**
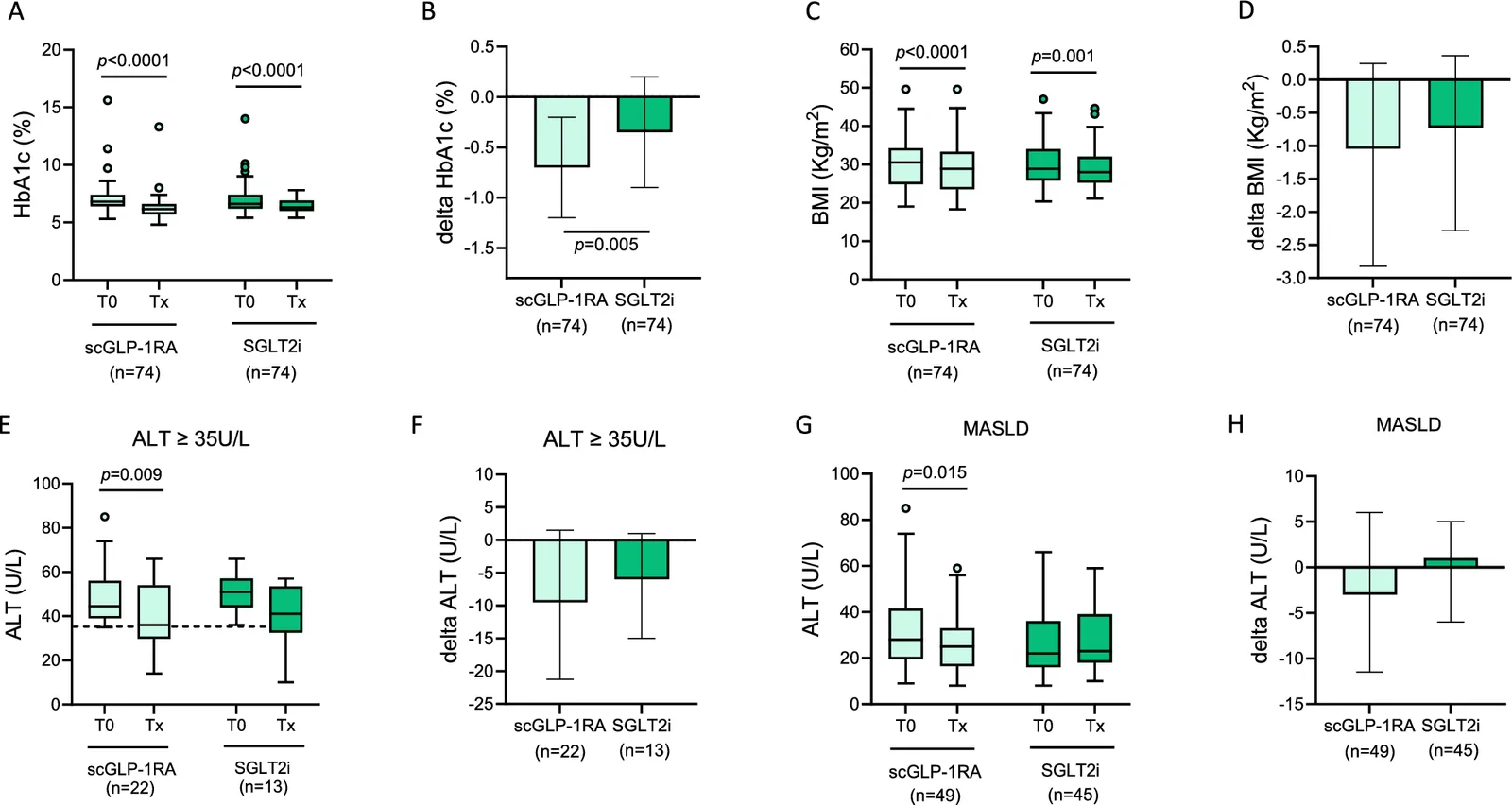
Therapeutic effects of scGLP-1RA and SGLT2i on metabolic and hepatic parameters.** (A)** Box-plots showing HbA1c reduction in both treatment groups over the 17-month follow-up period. **(B)** Comparison of median HbA1c reduction between the scGLP-1RA and SGLT2i groups, showing a significantly greater decrease in the scGLP-1RA cohort (p = 0.005). **(C)** Box-plots showing body mass index (BMI) reduction in both treatment groups over the 17-month follow-up period. **(D)** Comparison of median BMI reduction between the scGLP-1RA and SGLT2i groups. **(E)** Box-plots showing ALT reduction in both treatment groups stratified by baseline transaminase levels (≥ 35 U/L), showing that only in the scGLP-1RA there was a significant reduction. **(F)** Comparison of median ALT reduction between the scGLP-1RA and SGLT2i groups. **(G)** Box-plots showing ALT reduction in both treatment groups stratified for the presence of MASLD, showing that only in the scGLP-1RA there was a significant reduction. **(H)** Comparison of median ALT reduction between the scGLP-1RA and SGLT2i groups. Data are presented as medians with interquartile ranges (IQR). *p-values* < 0.05 are considered statistically significant

In order to compare scGLP-1RA to SGLT2i we performed a propensity score matching (PSM) analysis, whereby we selected 74 patients treated with scGLP-1RA and 74 patients treated with SGLT2i with no difference in terms of age, sex, baseline BMI and HbA1c (Table 2). In these patients, we evaluated the therapeutic response within each treatment group and then we compared the effects of the two drug classes. As shown in Fig. 1A-B, both scGLP-1RA and SGLT2i significantly reduced HbA1c from baseline. However, scGLP-1RA treatment was more effective in lowering HbA1c compared to SGLT2i. Specifically, the median reduction in HbA1c was − 0.7% (IQR − 1.2; − 0.20) in the scGLP-1RA group, whereas it was − 0.35% (IQR − 0.90; 0.20) in the SGLT2i group (p = 0.005). Both scGLP-1RA and SGLT2i significantly reduced BMI from baseline, with a median reduction of − 1.04 kg/m^2^ (IQR − 2.83; 0.24) in the scGLP-1RA cohort and − 0.73 kg/m^2^ (IQR − 2.29; 0.37) in the SGLT2i cohort, as shown in Fig. 1C-D. Regarding liver enzymes (Fig. 1E-H), ALT levels were significantly reduced only within the scGLP-1RA group, specifically in patients with baseline transaminases ≥ 35 U/L (p = 0.009) or with MASLD (p = 0.015). In contrast, no significant reduction in ALT was observed in the SGLT2i-treated cohort. Otherwise, AST and FIB-4 did not change. Table 3 summarizes the metabolic and hepatic parameters before and after treatment.

**Table 3:** Characteristics of the patients selected with PSM at baseline and after the treatment period

Variables	scGLP-1RA (N = 74)			SGLT2i (N = 74)			
	Baseline	After treatment	p-value	Baseline	After treatment	p-value	*p-value
BMI (kg/m^2^)	30.5 (24.9; 34.3)	28.8 (23.6; 33.1)	** < 0.0001**	28.9 (25.8; 33.8)	28.0 (25.4; 32.0)	**0.001**	0.680
HbA1c (%)	6.8 (6.4; 7.4)	6.1 (5.7; 6.6)	** < 0.0001**	6.6 (6.2; 7.4)	6.3 (6.0; 6.9)	** < 0.0001**	0.014
AST (IU/L)	21 (18; 26)	22 (18; 26)	0.283	21 (16; 27)	22 (16; 29)	0.077	0.690
AST ≥ 35U/L (IU/L),	41 (38; 56)	42 (33; 47)	0.282	46 (44; 52)	35 (30; 46)	0.113	0.835
AST (MASLD) (IU/L)	22 (18; 28)	22 (18; 26)	0.958	22 (17; 30)	23 (19; 30)	0.687	0.392
ALT (IU/L)	23 (15; 39)	22 (14; 33)	0.441	22 (16; 30)	21 (16;32)	0.950	0.957
ALT ≥ 35U/L (IU/L)	45 (39; 56)	36 (30; 54)	**0.009**	51 (44; 57)	41 (33; 54)	0.067	0.539
ALT (MASLD) (IU/L)	28 (20; 42)	25 (17; 33)	**0.015**	22 (16; 36)	23 (18; 39)	0.678	0.484
FIB-4 score	1.38 (1.03; 2.15)	1.34 (0.95; 1.79)	0.074	1.50 (1.13; 2.00)	1.46 (1.12; 2.08)	0.052	0.067

Data are reported as median and IQR. The p-values refer to the comparison between baseline and after treatment values within each group. The *p-values in the last column refer to the between-group comparisons after treatment. AST/ALT ≥ 35U/L indicates that groups were stratified by baseline transaminase levels ≥ 35U/L; AST/ALT (MASLD) indicates that groups were stratified by the presence of MASLD.

BMI, body mass index; HbA1c, glycated haemoglobin; AST, aspartate aminotransferase; ALT, alanine aminotransferase; MASLD, Metabolic Dysfunction-Associated Steatotic Liver Disease; FIB-4, Fibrosis-4 index

### Polymorphism distribution

Genetic analysis was performed on all 230 patients for *PNPLA3* rs738409 and *GLP1R* rs6923761 and rs761387 gene variants. As shown in Table 4, the *PNPLA3* rs738409 variant (MAF 30%) showed a distribution consistent with a T2DM population (p = 0.871)[19], despite differing from the general European population (p = 0.0001). Regarding the *GLP1R* rs6923761 variant, the minor allele frequency (MAF) was 27% and the distribution was in line with the general European population (p = 0.061). For the *GLP1R* rs761387 variant (MAF 12%), the distribution differed from the general population (p = 0.049) but was perfectly aligned with that expected for patients with T2DM or at metabolic risk (p = 0.715) [20].

**Table 4:** Distribution of allele frequencies

SNP	Nucleotide changes	Genotype	N (%)	MAF%	pHWE
*PNPLA3* *rs738409*	NM_025225.3:c.444C > G	CC	114 (49.6%)	30%	0.0001^a^ 0.871^b^
		CG	95 (41.3%)		
		GG	21 (9.1%)		
*GLP1R* *rs6923761*	NM_002062.5:c.502G > A	GG	123 (53.5%)	27%	0.061^a^
		GA	90 (39.1%)		
		AA	17 (7.4%)		
*GLP1R* *rs761387*	NM_002062.5:c.884 + 43A > G	AA	179 (77.8%)	12%	0.049^a^ 0.715^c^
		AG	45 (19.6%)		
		GG	6 (2.6%)		

MAF, minor allele frequency; pHWE, p-value of the χ2 test for Hardy–Weinberg equilibrium

^a^ as compared to the European population

^b^ as compared to T2DM patients[19]

^c^ as compared to T2DM patients[20]

The impact of all polymorphisms was analyzed using a dominant inheritance model. Regarding the *PNPLA3* rs738409 variant, 114/230 patients (49.6%) carried the wild-type CC genotype, while 95/230 patients (41.3%) were heterozygous (CG), and 21/230 patients (9.1%) were homozygous mutated (GG). For the *GLP1R* rs6923761 variant, 123/230 patients (53.5%) were wild-type (GG), 90/230 (39.1%) were heterozygous (GA), and 17/230 (7.4%) were homozygous mutated (AA). Finally, for the *GLP1R* rs761387 variant, 179/230 patients (77.8%) carried the wild-type AA genotype, 45/230 (19.6%) were heterozygous (AG), and 6/230 (2.6%) were homozygous mutated (GG).

### Impact of drug choice and genetic variants on metabolic response

Multivariate linear regression analysis was performed to evaluate if drug choice or genetic variants had an independent impact on HbA1c, BMI, ALT and AST reduction (Table 5). In particular, although the PSM balanced most clinical features, a significant difference remained for diabetes duration, which was therefore included as a covariate in all multivariable regression models to adjust for its confounding effect.

**Table 5:** Linear regression analysis

Predictive variable	HbA1c reduction			BMI reduction		
	β-estimate	95% CI	p-value	β-estimate	95% CI	p-value
Age	-0.002	[-0.013; 0.008]	0.633	0.041	[0.007; 0.075]	0.018
Sex (Male)	0.191	[-0.005; 0.386]	0.056	-0.217	[-0.877; 0.443]	0.517
SGLT2i (vs scGLP-1RA)	-0.349	[-0.555; -0.142]	**0.001**	-0.711	[-1.408; -0.015]	**0.045**
Baseline BMI	0.014	[-0.001; 0.029]	0.066	0.118	[0.067; 0.169]	** < 0.001**
Baseline HbA1c	0.745	[0.682; 0.809]	** < 0.001**	0.105	[-0.111; 0.320]	0.339
Diabetes duration	-0.018	[-0.031; -0.004]	**0.011**	-0.021	[-0.067; 0.025]	0.375
PNPLA3 rs738409	0.050	[-0.135; 0.235]	0.596	0.037	[-0.587; 0.661]	0.907
GLP1R rs761387	-0.277	[-0.506; -0.048]	**0.018**	-0.180	[-0.951; 0.592]	0.646
GLP1R rs6923761	0.027	[-0.166; 0.220]	0.781	0.132	[-0.519; 0.783]	0.690

Predictive Variable	ALT reduction			AST reduction		
	β-estimate	95% CI	p-value	β-estimate	95% CI	p-value
Age	-0.156	[-0.355; 0.042]	0.122	-0.152	[-0.278; -0.026]	0.018
Sex (Male)	2.352	[-1.494; 6.197]	0.229	0.041	[-2.407; 2.490]	0.973
SGLT2i (vs scGLP-1RA)	-1.409	[-5.466; 2.649]	0.494	1.366	[-1.217; 3.949]	0.298
Baseline BMI	0.330	[0.035; 0.625]	**0.029**	0.265	[0.077; 0.453]	**0.006**
Baseline HbA1c	0.645	[-0.609; 1.900]	0.312	0.344	[-0.455; 1.142]	0.397
Diabetes duration	-0.067	[-0.336; 0.201]	0.621	0.122	[-0.048; 0.293]	0.159
PNPLA3 rs738409	-1.041	[-4.677; 2.595]	0.573	-0.597	[-2.912; 1.718]	0.612
GLP1R rs761387	-0.566	[-5.060; 3.928]	0.804	0.110	[-2.751; 2.971]	0.940
GLP1R rs6923761	0.408	[-3.384; 4.201]	0.832	1.092	[-1.322; 3.507]	0.374

BMI, body mass index; HbA1c, glycated haemoglobin; AST, aspartate aminotransferase; ALT, alanine aminotransferase; GLP1R, glucagon-like peptide 1 receptor; PNPLA3: Patatin-like phospholipase domain-containing protein 3

The analysis showed that patients with higher baseline HbA1c had higher HbA1c reduction, while patients on SGLT2i (vs scGLP-1RA), or with longer history of T2DM had lower HbA1c reduction (β = -0.349, p = 0.001 for SGLT2i; β = -0.018, p = 0.011 for disease duration). In addition, the presence of* GLP-1R* rs761387 gene variant was associated with a minor reduction in HbA1c (β =  − 0.277, p = 0.02).

When looking at BMI reduction, the use of SGLT2i (vs scGLP-1RA) was associated with a lower BMI reduction (β =  − 0.711, p = 0.04), while older age and higher baseline BMI were significantly associated with greater BMI reduction during follow-up.

Finally, the reduction in liver transaminases was primarily driven by baseline BMI (β = 0.330, p = 0.029 for ALT; β = 0.265, p = 0.006 for AST), whereas age was an independent predictor only for AST reduction (β = -0.152, p = 0.018). Notably, the drug choice or the genetic variants studied did not independently influence the degree of transaminase reduction in the multivariable models.

## Discussion

The management of patients with T2DM and MASLD remains a clinical challenge, requiring therapies that address both glycemic control and hepatic health [6, 21]. Of note, in our cohort, the prevalence of hepatic steatosis was 67%, a finding that is highly consistent with the data of a large multicenter Italian study of patients with type 2 diabetes [22]. That being said, our study provides real-world evidence on the comparative efficacy of scGLP-1RA and SGLT2i in T2DM with MASLD and it highlights the pivotal role of genetic background in modulating treatment response.

When comparing the effects of SGLT2i to scGLP-1RA, our results demonstrate a superior biochemical impact of scGLP-1RA on metabolic parameters. In particular, the use of scGLP-1RA was associated with a greater HbA1c reduction after 1 year of treatment. This is consistent with the literature, which shows that SGLT2i can reduce HbA1c by 0.5–0.9%, while scGLP-1RA can reduce it by 0.9–2.2% [23]. In addition, in our study, the paired analysis of liver transaminases in patients with baseline ALT > 35 U/L or with MASLD showed that only scGLP-1RA were able to significantly reduce ALT levels after 1 year of treatment. This is consistent with the network meta-analysis by Gu et al., who identified semaglutide as the most effective agent for reducing ALT levels compared to dapagliflozin, with reductions of -14.7 U/L (95%CI -24.79, -4.61) vs -9.94 U/L (95%CI -18.42, -1.46) respectively [24]. Of note, Morieri et al. reported that the use of both SGLT2i and scGLP-1RA was associated with a significant reduction in ALT [22], although in their work it was not possible to perform a direct comparison between the two classes of drugs because patients had different baseline characteristics. Our linear regression analysis shows that the main drivers of liver damage and protection remain weight gain and loss, suggesting that the beneficial effect of scGP-1RAs on transaminases that we observed might have been due primarily to BMI reduction. However, it must be acknowledged that scGLP-1RA seem to exert direct beneficial effects on liver transaminases that are independent of BMI, likely through early actions on hepatocytes [25], as well as on Kupffer cells [26]. In line with this, recent data using mouse models of MASH have revealed also an endothelial cell-specific, weight-loss-independent, semaglutide-regulated, and GLP-1R-dependent intrahepatic network that acts locally to improve liver health [27]. Nevertheless, our results are therefore in line with current clinical recommendations, which suggest that scGLP-1RA or dual GIP/GLP-1RA should be the preferred therapeutic option in patients with T2DM and MASLD with moderate fibrosis or MASH, while SGLT2i can be taken into account in cases of MASLD with little or no fibrosis [6].

Given the existence of an inter-individual variability in treatment response, which has been attributed also to the patient’s genetic background, in this study we evaluated the impact of two *GLP-1R* polymorphisms and on the rs738409 polymorphism of the *PNPLA3* gene, which is recognized as the strongest genetic predictor of MASLD severity and progression [1, 12]. Our data show that the rs761387 polymorphism of the *GLP1R* was inversely associated with HbA1c reduction after 1 year of treatment (β = -0.277, p = 0.018). This is consistent with the study by Dorsey-Trevino et al. reporting that the rs761387 A > G variant was associated with glucose metabolism, as carriers of the minor G allele showed elevated postprandial glucose and GLP-1 levels [16]. By contrast, this data differ from our earlier observation that *GLP1R* polymorphisms did not impact on oral semaglutide response [18]. This could be due to the fact that in our previous work [18] patients were treated with oral semaglutide and/or to the entity of HbA1c reduction, as oral semaglutide reduced it by -0.3%, while in this study SGLT2i reduced it by -0.35% and scGLP-1RA reduced it by -0.7%.

Finally, we discuss the strengths and limitations of this study. Observational studies can complement the information provided by RCTs, as they offer insights as to whether an intervention is effective and safe in day-to-day clinical practice in a more heterogeneous patient group [28]. A major strength of this study is the head-to-head comparison between scGLP-1RA and SGLT2i cohorts, which was balanced through a Propensity Score Matching (PSM) analysis. This methodological approach is key in a real-world study comparing these two drug classes, which tend to be prescribed to patients with distinct clinical profiles. For instance, SGLT2i are frequently preferred for older patients with established cardiovascular or chronic kidney disease, while scGLP-1RA are typically initiated in younger individuals with higher BMI and more pronounced hyperglycemia, which is what we found in our population. Furthermore, the integration of pharmacogenetic data with real-world clinical outcomes represents an additional strength, allowing for a more granular understanding of inter-individual variability in treatment response. However, some limitations must be acknowledged. First, the retrospective nature of the study inherently carries risks of unmeasured confounding (for instance lifestyle was not taken into account), although PSM was used to mitigate this. In addition, due to the retrospective nature of the study, we did not evaluate changes in waist circumference, which adds significant information on visceral adiposity, or other liver parameters and liver ultrasound. Second, our study population exhibited relatively good glycemic control at baseline, with a median HbA1c of 6.8%, and this baseline profile may have partially limited the observable magnitude of drug effects or genetic variants. The assessment of MASLD was based on non-invasive biochemical markers and ultrasound, rather than liver biopsy, which remains the gold standard for diagnosing MASH and staging fibrosis. Finally, the follow-up period of 17 months, while sufficient to observe significant metabolic and enzymatic changes, may be too short to fully capture the long-term impact of these therapies.

In conclusion, our results support the preferential use of scGLP-1RA over SGLT2i for the management of hyperglycemia and weight loss in patients with MASLD. These observations are consistent with current clinical recommendations advocating for GLP-1RA or dual GIP/GLP-1RA as preferred options in this population [6]. SGLT2i might represent a valuable therapeutic alternative, particularly for patients with MASLD with little or no fibrosis and coexisting heart failure or renal impairment [6]. In addition, the association between *GLP1R* rs761387 variant and HbA1c reduction sheds light on the impact of this variant on treatment response. Further studies are needed to validate these genetic predictors in broader populations.

## Data Availability

The raw data supporting the conclusions of this article will be made available by the authors upon reasonable request.
